# Rotational and steric effects in water dissociative chemisorption on Ni(111)

**DOI:** 10.1039/c7sc02659e

**Published:** 2017-07-26

**Authors:** Bin Jiang

**Affiliations:** a Department of Chemical Physics , University of Science and Technology of China , Hefei 230026 , China . Email: bjiangch@ustc.edu.cn

## Abstract

Powerful laser techniques have recently enabled quantum-state resolved molecular beam experiments for investigating gas-surface reactions, which have unveiled intriguing vibrational, rotational, and also steric effects. For reactions involving polyatomic molecules, *e.g.*, the dissociative chemisorption of methane and water, the rotational and related steric effects are far less understood despite a large body of theoretical work having been able to reproduce the observed vibrational mode specificity and related bond selectivity semi-quantitatively or even within chemical accuracy. Herein, we report a high dimensional quantum dynamics study of water dissociation on Ni(111) on a first-principles potential energy surface, focusing on the reactivities of D_2_O in various rotational quantum states with different spatial orientations. Through an accurate quantum mechanical description of this asymmetric top, remarkable dependence of the reactivity on the orientation is observed. This dependence is site specific and rotational state specific. These single site rotational and steric effects are partially justified by a sudden model on the basis of the overlap between the rotational wavefunctions and the angular potential near the transition state, but rotational steering also plays a significant role which complicates the dynamics. Although site averaging weakens the influence of initial rotational excitations and leads to minor effects to the reactivity, steric effects are predicted to be observable if the water molecule is selectively excited and aligned by a linearly polarized laser.

## Abstract 2

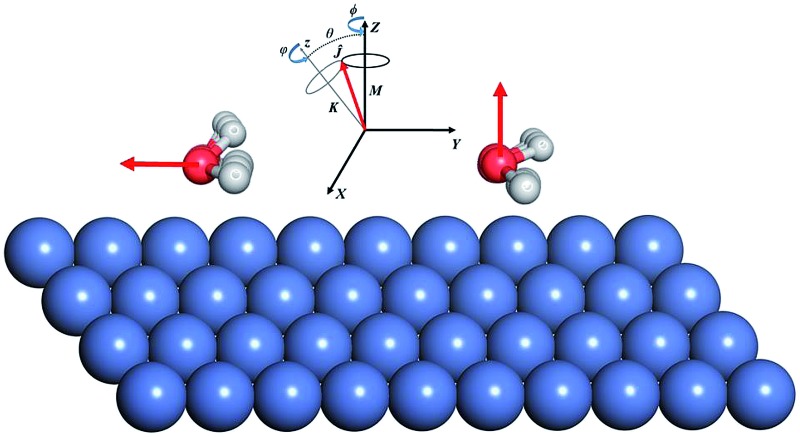
Weak rotational but significant steric effects are predicted in water dissociative chemisorption on Ni(111).

## I. Introduction

How the translational, vibrational and rotational energy, and the orientation of reagents influence chemical reactivity is of central importance in chemical reaction dynamics.^1^ In gas-surface reactions, the surface itself is already well defined in the laboratory frame, and the gaseous molecules can now be exquisitely controlled with state-of-the-art molecular beam and laser techniques.^2,3^ Consequently, they provide a unique opportunity to answer these key questions. Indeed, the effects of translational energy and vibrational excitations on the dissociative chemisorption (DC) of methane and water (including their isotopologues) on various metal surfaces have been extensively examined,^4–18^ providing unprecedented dynamical details at the single quantum state level. Stimulated by these intriguing experiments, persistent theoretical efforts have been made towards gaining an in-depth understanding of the multi-dimensional potential energy surface (PES) and dissociation dynamics.^19–32^ These studies have qualitatively or semi-quantitatively reproduced the experimentally observed mode specificity and the bond selectivity, as well as lattice effects.^33–36^ Furthermore, a simple transition-state based model has been proposed to explain the vibrational mode specific and bond selective phenomena in dissociative chemisorption.^37^

Rotational and steric effects are often more subtle not only because of the much smaller energy gap between rotational states (which may even be degenerate), but also because they rely on fine angular anisotropy along the reaction pathway thus representing a more sensitive probe for the global PES.^38^ For example, it has been well established that the “helicopter” type of rotation (*m*_j_ = *j*) is more favorable than the “cartwheel” one (*m*_j_ = 0) for H_2_ dissociation on a copper surface because the H–H bond lies parallel to the surface at the transition state, representing strong alignment effects.^39–44^ In this regard, the rotational and steric effects in the DC of polyatomic molecules on metal surfaces are poorly understood due to their complexity. Experimentally, in practice, the use of infrared (IR) lasers mostly pumps the reactant to a ro-vibrationally excited state. The Utz and Beck groups have independently found relatively minor rotational effects of CH_4_(*ν*_3_ = 1, *J* = 0–3) dissociation on nickel.^45,46^ Beck and coworkers have further discovered that the reactivity changes as much as 60% with the initial alignment of the ro-vibrationally excited CH_4_ and CHD_3_ with respect to the surface.^47,48^ Theoretically, although neither the reduced dimensional quantum dynamical nor the full-dimensional *ab initio* molecular dynamics (AIMD) calculations for the CHD_3_ + Pt(111) system reproduced the observed steric effects,^32,49^ it was suggested that the rotational degrees of freedom (DOFs) should be included in a dynamical model.^49^ Our recent quasi-classical trajectory (QCT) results for CHD_3_ dissociation on Ni(111) based on a twelve-dimensional PES^23^ qualitatively reproduced the experimental polarization angle dependence of the dissociation probability, indicating the importance of treating the probability distribution as a function of the polarization angle quantum mechanically.^50^ A quantitative understanding of the rotational and steric effects requires a fully coupled description of the ro-vibrational state of methane, involving at least 13 DOFs (neglecting lateral coordinates) and being (2*J* + 1) times more expensive than the ground state calculation. Such calculations are still intractable.

Another representative but simpler system is the DC of water, which is not only the rate-determining step in the water gas shift (WGS) reaction^51^ but also an essential step in steam methane reforming.^52^ Since we reported the first six-dimensional PES and QD model for water dissociation on a rigid Cu(111) surface,^53^ a great number of theoretical models have emerged and they have concentrated on the mode specificity and bond selectivity of water DC on various metal surfaces.^53–64^ On the other hand, the quantum state resolved experiment by Beck and coworkers has confirmed the significant enhancement of reactivity on Ni(111) by exciting the D_2_O molecule to its ro-vibrationally excited states, *i.e.* D_2_O(1*ν*_3_, *J*_*K*_a_*K*_c__ = 2_12_) and D_2_O(2*ν*_3_, *J*_*K*_a_*K*_c__ = 3_13_),^65^ which, however, has not been quantitatively understood yet. One of the possible issues is that rotational excitations were not considered in all previous studies except for two crudely approximate reduced dimensional models.^66,67^ Very recently, nine-dimensional (9D) PESs for water DC on rigid Ni(111) and Cu(111) have become available^59,64^ and fixed-site seven-dimensional (7D) QD calculations^58–61^ have been able to accurately reproduce the QD results involving all nine molecular DOFs^57^ *via* a well tested site-averaging scheme.^68^ These works offer the opportunity for rigorous quantum mechanical treatment of the rotation and orientation of water as an asymmetric top that is difficult to describe classically, which is the goal of our work here. This article is structured as follows: Section II describes the methodology. Section III presents and discusses the site-specific and site-averaged nine-dimensional rotational and orientational effects. Concluding remarks are given in Section IV.

## II. Theory

**Fig. 1:**
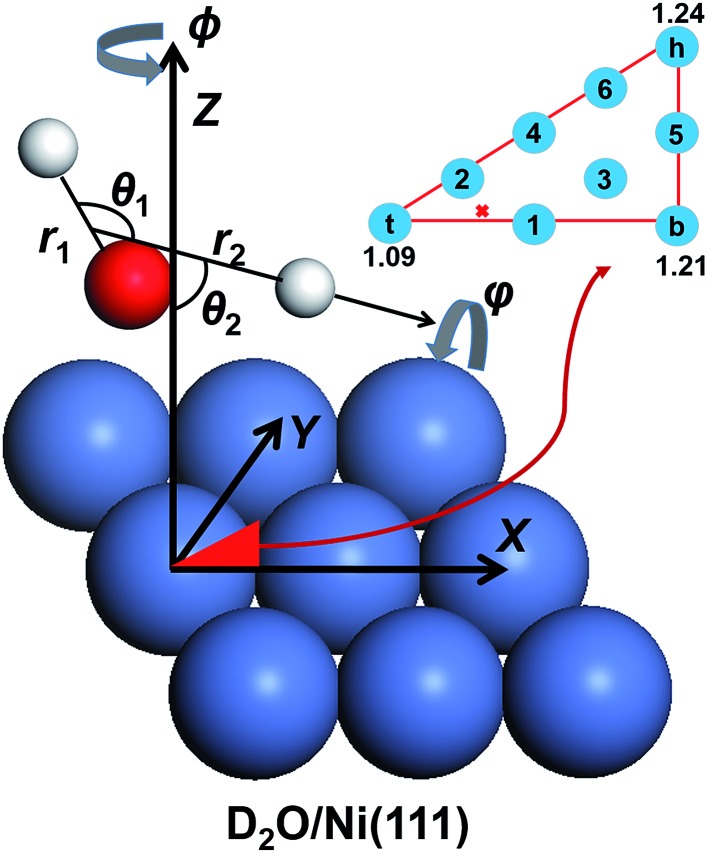
The coordinate system used in the quantum dynamical calculations and the selected nine sites for averaging in the irreducible triangle on the Ni(111) facet (inserted in the upper right corner). The site-specific barrier heights (in eV) are marked for the top, bridge and hollow sites. The global transition state is labeled as a red cross.

In the present work, we computed the initial ro-vibrationally state selected reaction probabilities in the 7D fixed-site model *via* a time-dependent wave packet method, and the site-averaging yielded the final 9D results in a good approximation. Following our previous work,^60^ the DC of water on a rigid surface is described in Fig. 1 with nine internal coordinates, and the fixed-site Hamiltonian can be expressed in the seven internal coordinates, with (*X*, *Y*) frozen above a specific site as (ℏ = 1 hereafter):1
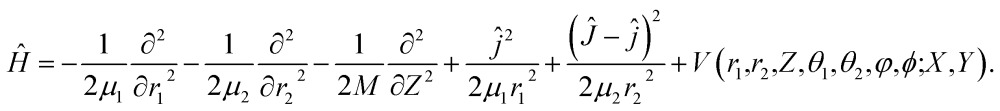



Here, the molecule that was studied in the experiment is D_2_O.^65^ *r*_1_ stands for the non-dissociative OD bond length, *r*_2_ is the distance between the center of mass of OD and D, *μ*_1_ = *m*_D_ *m*_o_/(*m*_D_ + *m*_o_) and *μ*_2_ = *m*_OD_ *m*_D_/(*m*_OD_ + *m*_D_) are the corresponding reduced masses, *M* is the total mass of D_2_O and *Z* is the height of the molecular center above the surface. *ĵ* and *Ĵ* are the angular momentum operators of OD and D_2_O, respectively. *V* is the 9D PES fitted to over twenty five thousand density functional theory (DFT) points computed by the RPBE functional,^69^ with the (*X*, *Y*) components fixed at specific values. The properties of this PES have been previously discussed in detail,^61^ so only brief descriptions are given here. The transition state locates near but not exactly on the top site, with the lowest barrier height being ∼1.03 eV. The dissociating O–H bond length is 1.59 Å at the transition state and the oxygen atom is above the surface at 1.98 Å, featuring an apparent “late” barrier. The surface corrugation leads to site specific barriers, *e.g.* the top, bridge and hollow sites correspond to the barrier heights of 1.09, 1.21 and 1.24 eV, respectively. Our earlier work suggested that this PES was in better agreement with the experiment for the vibrationally excited states.^61^

The 7D time-dependent wave packet is expanded in terms of the radial and rotational basis functions,2
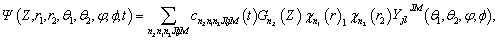
where the overall rotational basis *Y*_*jl*_^*JM*^(*θ*_1_,*θ*_2_,*φ*,*φ*) is expressed as,3
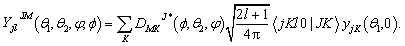



Here, *D*_*MK*_^*J*^(*φ*,*θ*_2_,*φ*) is the Wigner rotation matrix, *y*_*jK*_(*θ*_1_,0) is the spherical harmonics, and *j*, *l* and *J* are the angular momentum quantum numbers associated with OD, the *r*_2_ (orbital) and D_2_O, respectively. *K* and *M* are the projections of *J* on the molecule-fixed *z* axis (*r*_2_) and the space fixed *z* axis (*i.e.* surface normal), respectively.

To study the rotational and orientational effects, the initial eigenfunction of D_2_O in its specific ro-vibrational state 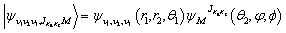 was obtained by diagonalizing the triatomic Hamiltonian with *Z* = ∞, where the rotational wave function of this asymmetric top 
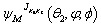
 is a linear combination of the Wigner rotation matrices. The initial wave packet was constructed as a product of this eigenfunction and a Gaussian wave packet centered in the asymptote *Z* = *Z*_0_, and propagated by the split-operator method.^70^ The initial state-specific dissociation probability (*P*_0_) was obtained by a flux analysis method after the transition state.^71^ The final 9D probability was well approximated by a weighted sum of 7D results at 9 selected sites,^57^ which are included in Fig. 1. All reaction probabilities displayed have been multiplied by a factor of two, accounting for the two equivalent OD bonds. More details about the PES and QD calculations and parameters can be found in ref. 60 and 61.

Unlike our earlier 6D flat surface model, it should be emphasized that the quantum number, *M*, which specifies the orientation of the molecule rotating with respect to the surface normal, is not conserved but associated with the anisotropic potential in *φ*. As a result, it is explicitly involved in the dynamics calculations, giving rise to a 2*J* + 1 fold degeneracy of each rotational state |*J*_*K*_a_*K*_c__ with *M* taking values from –*J* to *J*, representing totally different spatial distributions. In the absence of an external field, the molecule possessed an average over all the possible *M* values, and the dissociation probability for a specific rotational state was averaged over all (2*J* + 1) projection states. However, the relative weights of various *M* components can be controlled by the laser polarization direction *via* excitation, as realized in a recent experiment by Beck and coworkers, who successfully aligned methane before impacting it on Ni surfaces and observed alignment dependent reactivity.^47,48^

## III. Results and discussion

**Fig. 2:**
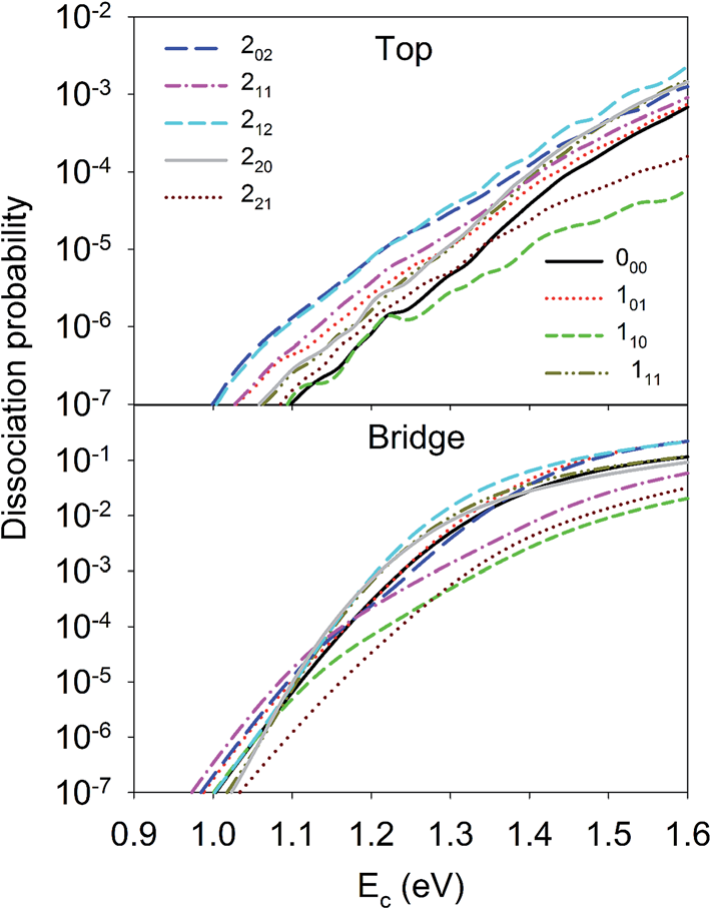
Dissociation probabilities as a function of collision energy of D_2_O(*ν* = 0, *J*_*K*_a_*K*_c__ up to *J* = 2, *M* = 0) at the top (upper panel) and bridge (bottom panel) sites.

Let us first discuss the site-specific dynamical results which reflect the original rotational and orientational effects that are not averaged and are relatively easy to analyze. The reaction probabilities for the initial rotational states *J* = 0, 1 and 2, with *M* = 0, are compared in Fig. 2. For this particular orientation, the total angular momentum, *J*, lies in the plane perpendicular to the surface normal. It is clear that the reaction probability sensitively depends on the rotational excitation and incident energy, as well as the impact site. This phenomenon is similar to that in our earlier 6D QD model describing H_2_O dissociation on Cu(111) with a flat 6D PES.^67^ It is shown that at the top site the 1_10_ and 2_21_ states generally decrease the reactivity compared with the ground state D_2_O, while other states, especially the 2_02_, 2_12_, and 1_01_ states, increase the reactivity. While the general trend does not change much at the bridge site, the 1_11_ and 2_20_ states, and especially the 2_11_ state, become less reactive. The dissociation probabilities at the hollow site are similar to these at the bridge site and are thus not shown. It should be noted that the bridge and hollow sites are much more reactive than the top site although the former’s barrier heights are obviously higher, which has been attributed to the effectiveness of converting the translational energy into the reaction coordinate.^64^ This effectiveness stems from the angle of the “elbow” shaped PES, where the larger the angle formed between the translational and reaction coordinates is, the more easily the translational energy can be transferred to the reaction coordinate to overcome the barrier, as has been shown in our previous work.^60,63,64^ In any case, the differences between the dissociation probabilities of these low-lying rotational states can be as large as an order of magnitude, which is significant given the very small energy differences between them, *i.e.* a few tens of cm^–1^. These results highlight the complex impact of the initial rotational excitation on the reactivity.

**Fig. 3:**
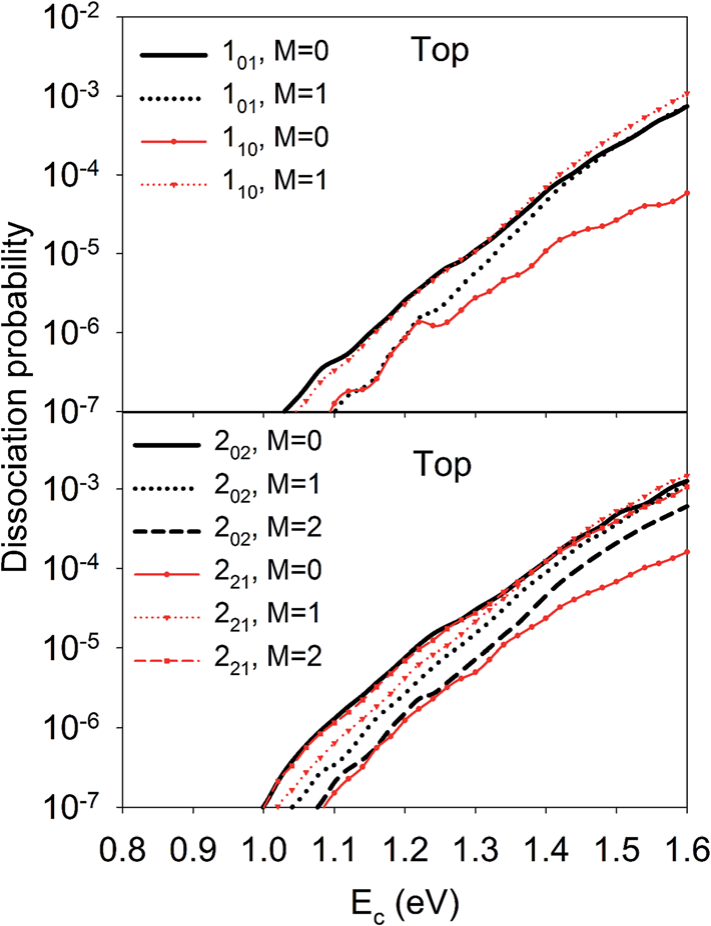
*M*-dependent dissociation probabilities as a function of collision energy of selected rotational states of D_2_O(*ν* = 0, *J*_*K*_a_*K*_c__) at top site.

On the other hand, the orientation of the D_2_O molecule was also found to significantly influence the site-specific reactivity. As shown in Fig. 3, at the top site, the 1_01_ and 1_10_ states presented the opposite dependence of reactivity on orientation, *e.g* the *M*= 0 component was more reactive for the 1_01_ state than *M* = 1, while less reactive for the 1_10_ state. Similarly, the dissociation probability of the 2_02_ state decreased with increasing *M*, and the opposite was true for the 2_21_ state. Such steric effects were also observed on other impact sites, though in a more complex fashion and dependent on the translational energy. Since *M* is the projection of the total angular momentum onto the space-fixed axis, *i.e.*, the surface normal here, *M* = 0 corresponds classically to a cartwheel-like rotation while *M* = *J* is helicopter-like. However, the distinct stereodynamics of the different rotational states displayed in Fig. 3 are difficult to rationalize in a classical picture which has been frequently invoked in the diatomic DC process. For example, in the activated H_2_ DC on Cu(111) and Ag(111) with notable barriers,^39–43,72^ it has been found that the “helicopter” type of rotation (*m*_j_ = *j*) is more favorable than the “cartwheel” one (*m*_j_ = 0) because the former can access the transition state with H_2_ lying parallel to the surface more easily.

**Fig. 4:**
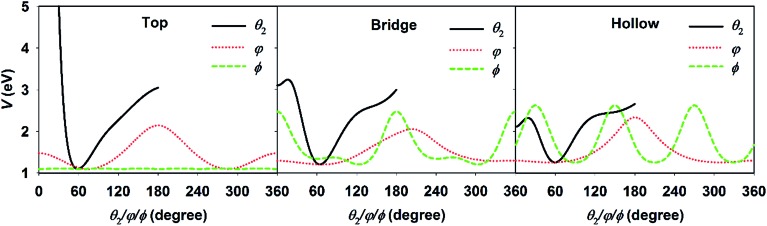
The angular potentials with respect to *θ*_2_, *φ*, and *φ*, with other coordinates fixed at the transition state geometries at the top (left), bridge (middle), and hollow (right) sites.

For the DC of a polyatomic molecule, especially an asymmetric top, it is shown above that the rotational and steric effects become more complicated. We have previously studied the rotational effects in polyatomic reactions in the gas phase, *e.g.* H + H_2_O and Cl + CHD_3_ reactions,^73,74^ where the reactivity is highly dependent on the correlation between the initial rotational wavefunction and the angular anisotropy in the potential energy landscape near the transition state. This concept also worked reasonably well in our previous 6D model of H_2_O DC on Cu(111) neglecting the azimuthal dependence of the PES.^67^ In the present work, three rotational angles were associated with the molecular rotation and orientation, *i.e.*, *θ*_2_, *φ*, and *φ*. Fig. 4 displays the PESs with respect to these three coordinates with other coordinates kept at the site-specific transition state values. The minimum of each curve corresponds to each coordinate at the transition-state geometry. It can be clearly seen that the PESs at the top, bridge, and fcc sites are all very tight in *θ*_2_ and roughly centered at ∼60°, but vary more weakly over a broad range in *φ* and *φ*. This is understandable as the *θ*_2_ largely determines the distance of the dissociating hydrogen atom above the surface. When the dissociating hydrogen atom moves too close to the surface, the repulsive force becomes strong; while when it moves too far away from the surface, the interaction between the hydrogen and surface atoms becomes weak. Both factors increase the potential energy. As a result, following our earlier work,^67,73^ we evaluated the so-called effective barrier defined as the integral: 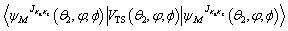, with *θ*_2_ ∈ [35°, 95°] and *V*_TS_(*θ*_2_, *φ*, *φ*), which are the three dimensional angular PESs with other coordinates fixed at the transition state. A basic assumption here is that the rotational wavefunction is unchanged as the molecule approaches the transition state, a small effective barrier thus implies that the wavefunction is largely populated around the barrier region, leading to a higher probability of overcoming the barrier and higher reactivity.

The effective barriers for the top site as an example are listed in Table 1, where the numbers are scaled so that the ground state value is unity. We noted that these barriers are similar at the bridge and hollow sites as well. There is a qualitative correlation between the dissociation probabilities and the effective barriers. For example, the effective barriers for the *M* = 0 component of the 1_01_, 2_02_, 2_12_ and 2_11_ states are lower than that of the ground state, consistent with the reactivity enhancement of these rotational states over the ground state in Fig. 2. On the other hand, the 1_10_ and 2_21_ states have higher effective barriers corresponding to lower reactivities in Fig. 2. In addition, the *M* dependent dissociation probabilities can also be qualitatively explained by the effective barriers. For instance, the 1_01_ and 2_02_ states have higher effective barriers as *M* increases, while the opposite is true for the 1_10_ and 2_21_ states, which agrees reasonably well with the trend of dissociation probabilities. Nonetheless, some other states do not obey the predictions made by this crude model. For example, both the 1_11_ and 2_20_ states with *M* = 0 show slight enhancement, however they have very high effective barriers. In addition, the 2_11_ state is more reactive than the ground state at the top site, but less reactive at the bridge site, despite its effective barrier being consistently smaller than that of the ground state at any site.

**Table 1:** Relative frequencies and effective barriers at the top site for low-lying rotational states of D_2_O with respect to the ground state

|*J*_*K*_a_*K*_c__, *M*	Frequency (cm^–1^)		Relative effective barriers (arb. unit)
	Calc.	Expt.^*a*^	
|0_00_, *M* = 0	0.00	0.00	1.00
|1_01_, *M* = 0/1	11.75	12.12	0.72/1.17
|1_11_, *M* = 0/1	19.52	20.26	1.15/0.92
|1_10_, *M* = 0/1	21.89	22.68	1.12/0.94
|2_02_, *M* = 0/1/2	34.79	35.88	0.85/0.88/1.19
|2_12_, *M* = 0/1/2	40.67	42.07	0.89/0.97/1.08
|2_11_, *M* = 0/1/2	47.76	49.34	0.87/0.96/1.10
|2_21_, *M* = 0/1/2	70.99	73.68	1.19/1.11/0.80
|2_20_, *M* = 0/1/2	71.46	74.14	1.21/1.08/0.81

^*a*^ Ref. 76.

**Fig. 5:**
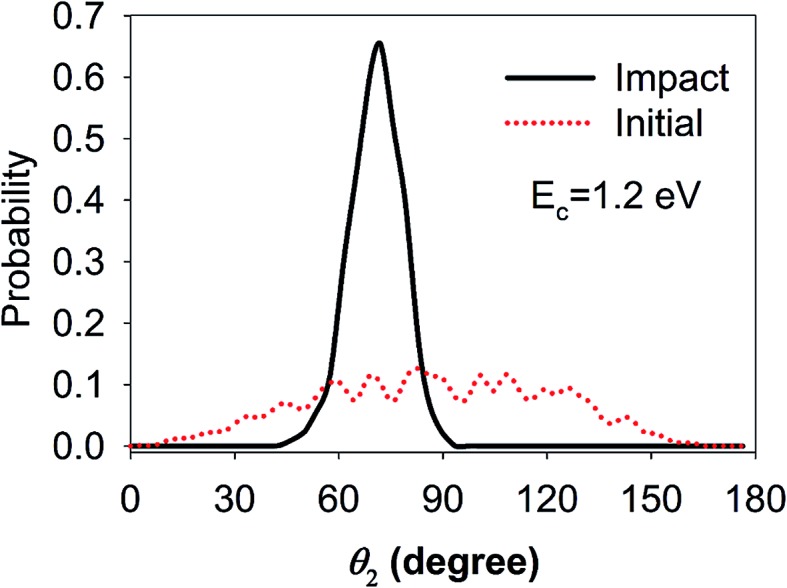
Polar angular distributions from quasi-classical trajectory calculations of ground state D_2_O with a collision energy of 1.2 eV. The distributions of the reactive trajectories in the beginning (red dotted line) and at the impact point (black solid line, *r*_OD_ is stretched to 1.5 Å) are shown.

These contradictions underscore the limitations of this “sudden” approximation, which excludes the rotational steering effect,^44^ *i.e.*, the molecule can be “guided” by the potential anisotropy to the transition state and the influence of the initial rotational excitation is lost when accessing the barrier. To investigate this possibility, QCT calculations have been carried out for the ground state D_2_O and the technical details can be found elsewhere.^64^ In particular, we monitored the most important polar angle (*θ*_2_) and plotted the polar angular distributions of the reactive trajectories in the beginning and when the O–D bond was elongated to 1.5 Å, as shown in Fig. 5. The collision energy was 1.2 eV, which is higher than the lowest barrier, and the distributions were found to not vary much with increasing collision energies. It is somewhat surprising that the rotational steering effect is so pronounced in this reaction. That is, the angular distribution of *θ*_2_ was very broad in the initial condition while it became quite narrow and peaked nearby the transition state geometry when the O–D bond started to dissociate, suggesting that the molecule reorientates to adapt the transition state geometry so as to avoid a higher bottleneck due to anisotropy. This phenomenon is in contrast with that of a similar system, *i.e.*, the DC of methane, which is a spherical rotor, on Ni(111), where rotational steering was found to be less important.^28,36^ As a consequence, although water DC is a direct process it is more rotationally adiabatic than sudden, and therefore the less satisfactory prediction by the “sudden” effective barrier is understandable. Such rotational adiabaticity may depend on the individual rotational state and impact site, which results in the complexity of the site-specific rotational effects.

**Fig. 6:**
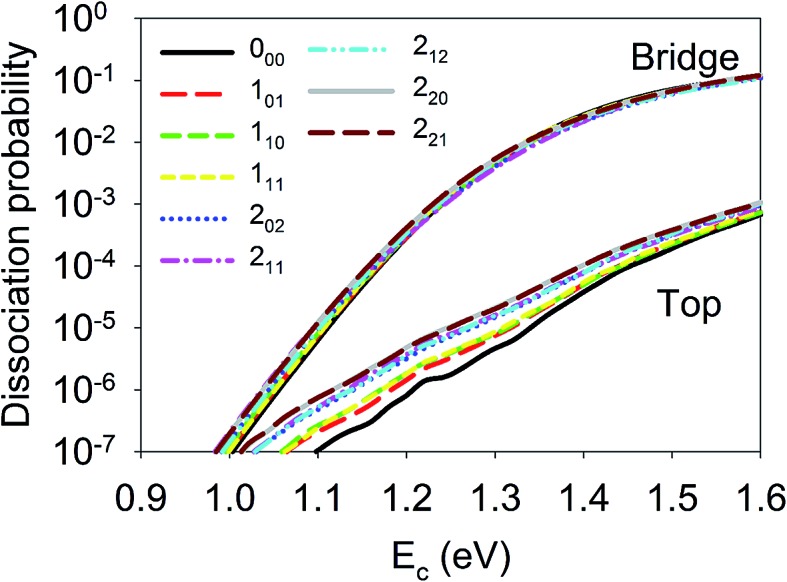
Dissociation probabilities as a function of collision energy of *M*-averaged D_2_O(*ν* = 0, *J*_*K*_a_*K*_c__ up to *J* = 2) at the top (upper panel) and bridge (bottom panel) sites.

**Fig. 7:**
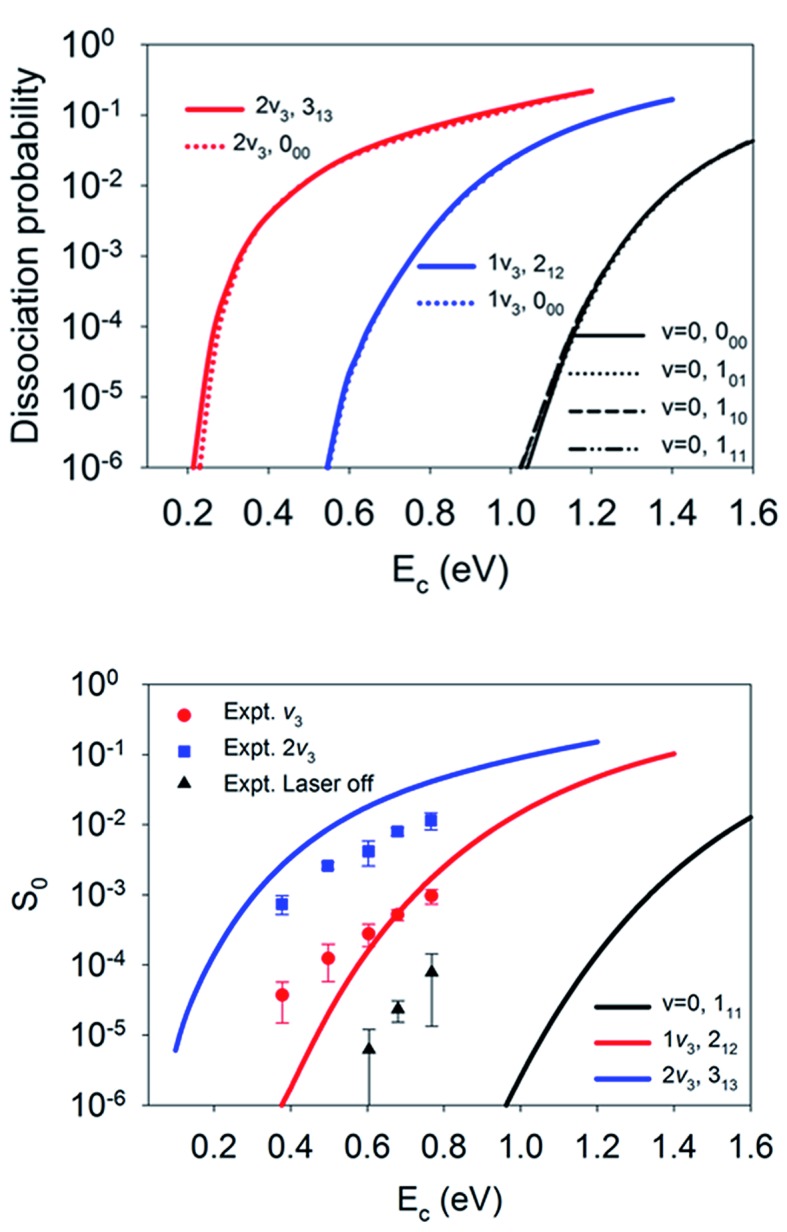
The upper panel displays site-averaged dissociation probabilities as a function of collision energy of the *M*-averaged D_2_O(*ν* = 0, 0_00_, 1_01_,1_10_, 1_11_), D_2_O(1*ν*_3_, 2_12_), and D_2_O(2*ν*_3_, 3_13_) states. The lower panel shows a comparison of sticking probabilities with the lattice effects corrected of the D_2_O(*ν* = 0, 1_11_), D_2_O(1*ν*_3_, 2_12_), and D_2_O(2*ν*_3_, 3_13_) states and available experimental data.^65^

Although the aforementioned site-specific analysis is useful for a better understanding of the mechanisms of rotational and steric effects, these results are not experimentally measurable. In most experiments, only the site and orientation averaged sticking probabilities can be measured. In Fig. 6, *M*-averaged dissociation probabilities at the top and bridge sites are compared, which were computed by 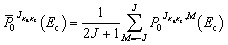. It is interesting that the orientation averaging weakens the influence of the overall rotational excitation as compared with that in Fig. 2, especially at the bridge site. In general, the reactivity seems to slightly increase with *J* and the efficacies of the (2*J* + 1) rotational states with the same *J* are more or less the same. This observation is analogous to those predicted in our previous 6D model and the 3D model of Tiwari *et al.* on Cu(111),^66,67^ while these less accurate approximations lead to more significant enhancement with an increasing rotation quantum number (*J*). Indeed, the *M*-averaged effective barrier of every single rotational state was closer to unity and varied more weakly than the *M*-dependent counterpart, which is consistent with the dynamical results. The residual differences in reactivity due to rotational excitations were further eliminated due to site-averaging, as demonstrated in Fig. 7. Here, the approximate nine-dimensional site-averaged dissociation probabilities for the vibrationally ground state and two asymmetric stretching (*ν*_3_) excited states are compared with and without rotational excitations. D_2_O(1*ν*_3_, 2_12_) and D_2_O(2*ν*_3_, 3_13_) are of interest because they were prepared in Beck’s experiment with excitation from D_2_O(*ν* = 0, 1_11_).^65^ Compared to the ground state, it is clear that the rotational excitations barely alter the dissociation probability. Interestingly, the lack of a pronounced rotational effect was also observed experimentally in CH_4_(*ν*_3_ = 1, *J* = 0–3) dissociation on nickel surfaces,^45,46^ which validates the commonly used rotationless model.^53,57–59,65^ These results indicate that both site and orientation averaging would wipe off the rotational effects. Indeed, as shown in the lower panel in Fig. 7, the calculated sticking coefficients D_2_O(*ν* = 0, 1_11_), D_2_O(1*ν*_3_, 2_12_) and D_2_O(2*ν*_3_, 3_13_) still differ from the experimental counterparts. This disagreement may be attributed to the inaccuracy of density functional. As a result, a more accurate density functional may be desired for a quantitative description of this reaction.^75^

**Fig. 8:**
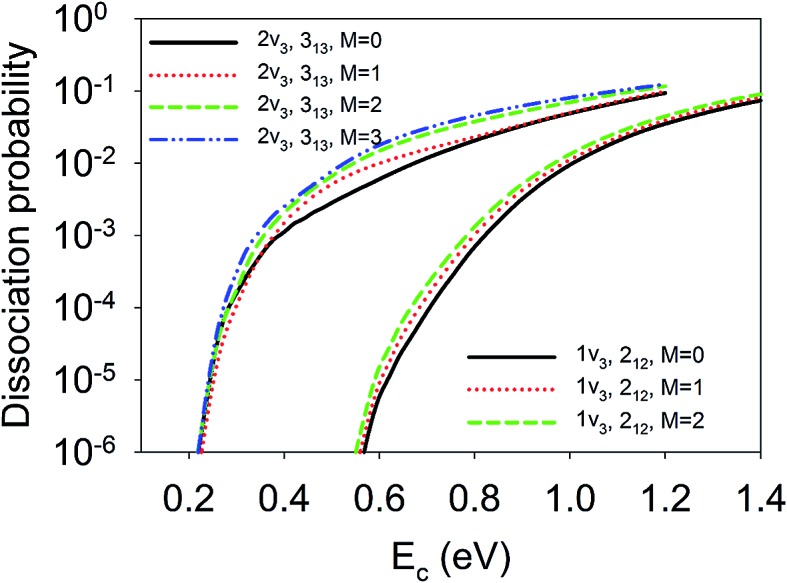
The site-averaged *M* dependent dissociation probabilities of the D_2_O(1*ν*_3_, 2_12_) and D_2_O(2*ν*_3_, 3_13_) states.

Finally, we show in Fig. 8 the site-averaged dissociation probabilities for vibrationally excited states with different orientations, which can be prepared experimentally with a linearly polarized laser. Such experiments have been done for methane dissociation on nickel surfaces by Beck and coworkers.^47,48^ As a result of contributions from all nine impact sites, for the 1*ν*_3_ and 2*ν*_3_ states, the site-averaged dissociation probabilities increased with *M*. In other words, the *M* = *J* state, rotating more or less like a “helicopter”, is more reactive than the *M* = 0 state, featuring a “cartwheel-like” rotation. For example, the dissociation probabilities at *E*_c_ = 0.8 eV for D_2_O(1*ν*_3_, 2_12_, *M* = 0/1/2) were 6.97 × 10^–4^, 1.03 × 10^–3^, and 1.33 × 10^–3^, respectively; while those at *E*_c_ = 0.6 eV for D_2_O(2*ν*_3_, 3_13_, *M* = 0/1/2/3) were 6.04 × 10^–3^, 1.00 × 10^–2^, 1.50 × 10^–2^, and 1.80 × 10^–2^, respectively. This means the reactivity can be increased twice or three times just by aligning the molecule with different branches of infrared transitions, *e.g.*, R(0) and Q(1).^47,48^ These steric effects are at least comparable to, and perhaps more remarkable than those measured by Beck and coworkers by controlling the initial alignment of the ro-vibrationally excited CH_4_ and CHD_3_ with respect to the surface.^47,48^ It is important to note that none of the previous reduced dimensional QD and AIMD results^32^ reproduced the experimental data of the Beck group on methane dissociation, not even the trend. Our results indicate that a full-dimensional treatment of the rotation and impact site may be essential to explain the experimentally observed subtle steric effects.

## IV. Concluding remarks

To summarize, we in the present work report a comprehensive quantum dynamical study on the rotational and steric effects of water dissociation on Ni(111). The 7D fixed-site QD calculations have revealed strong rotational and orientational dependence of the reactivity, which is partially understood by the angular anisotropic barriers with respect to molecular rotation near the transition state, but further complicated by rotational steering. However, averaging over all of the possible orientations and the impact sites gives rise to an almost negligible influence of the rotational excitation, which is consistent with the experimental observations of methane dissociative chemisorption on Ni(100). More importantly, the approximate nine-dimensional results suggest that the steric effects still exist and the *M* = *J* orientation is more reactive than the *M* = 0 orientation for both the D_2_O(1*ν*_3_, 2_12_) and D_2_O(2*ν*_3_, 3_13_) states. These predictions are sufficiently large to be observable in experiment by controlling the alignment of the water molecule with selected infrared transitions, as has been done for CH_4_/CHD_3_ dissociation on several Ni surfaces, but which has not yet been fully understood.^47,48^ It is hoped that the results presented here will stimulate new experiments to shed light on, and lead to a more complete understanding of, the rotational and steric effects in the DC of polyatomic molecules.
